# Supplementary material for: Somatostatin analog therapy effectiveness on the progression of polycystic kidney and liver disease: A systematic review and meta-analysis of randomized clinical trials
Source: PLoS One. 2021 Sep 24;16(9):e0257606. doi: 10.1371/journal.pone.0257606 (PMC8462725; doi:10.1371/journal.pone.0257606)
Supplement: S1 Fig — (DOC) [file pone.0257606.s001.doc]

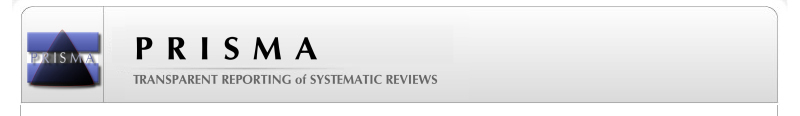
**(S1 Fig) PRISMA 2009 Flow Diagram**

**Screening**

**Included**

**Eligibility**

**Identification**

Records identified through database searching between January 2018 and November 2020

(n = 62)
(n = 435)

Records after duplicates removed
(n = 264)

Records screened
(n = 264)

Records excluded (n = 215)

-Not original research (n = 144)

-Not RCT (n= 35)

-Without our interest outcomes (n= 12)

-Case reports (n=11)

-Animal studies (n=7)

-Overlapping with other studies (n=6)

-Journal protocol (n=2)

Full-text articles assessed for eligibility
(n = 49)

Full-text articles excluded, with reasons (n = 36)

-Not original research (n= 14)

-Overlapping with other studies (n=8)

-Without our interest outcomes (n= 5)

-Not RCTs (n=5)

-Case reports (n= 4)

Studies included in qualitative synthesis
(n = 13)

Studies included in quantitative synthesis (meta-analysis)
(n = 10)

Full-text articles excluded, with reasons (n = 3)

-Overlapping with other studies (n=2)

-Insufficient information (n=1)

Records identified through database searching up to May 2018
(n = 350)
