# Supplementary figures and images for: Somatostatin analog therapy effectiveness on the progression of polycystic kidney and liver disease: A systematic review and meta-analysis of randomized clinical trials
Source: PLoS One. 2021 Sep 24;16(9):e0257606. doi: 10.1371/journal.pone.0257606 (PMC8462725; doi:10.1371/journal.pone.0257606)

**(S2 Fig) Risk of bias assessment**

**Risk of bias summary**


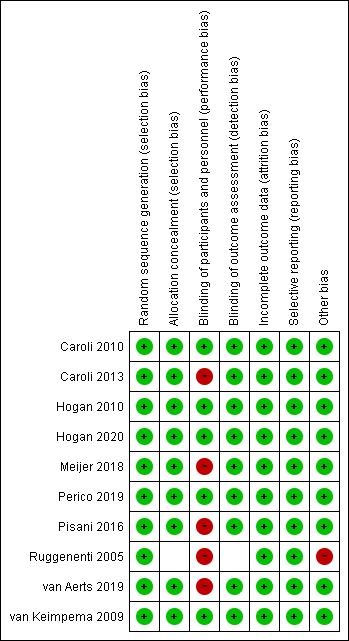


**Risk of bias graph**


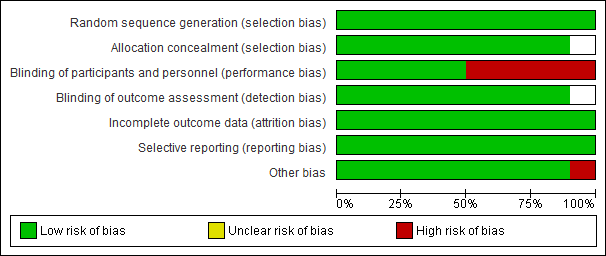

Supplement: S2 Fig — (DOCX) [file pone.0257606.s002.docx]
