# Supplementary material for: Somatostatin analog therapy effectiveness on the progression of polycystic kidney and liver disease: A systematic review and meta-analysis of randomized clinical trials
Source: PLoS One. 2021 Sep 24;16(9):e0257606. doi: 10.1371/journal.pone.0257606 (PMC8462725; doi:10.1371/journal.pone.0257606)

**(S3 Fig) Sensitivity Analysis**

(S3 - 1) Sensitivity analysis of TLV


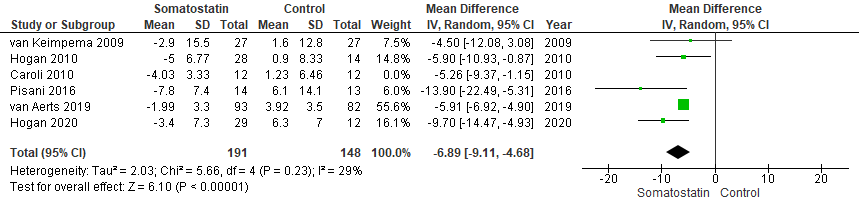


(S3 - 2) Sensitivity analysis of TKV


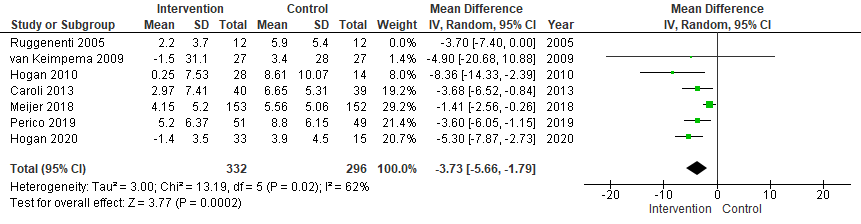


(S3 - 3) Sensitivity analysis of eGFR


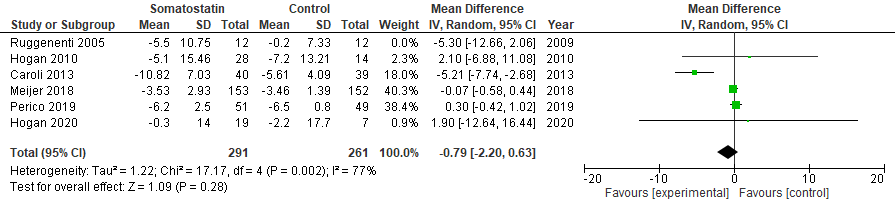

Supplement: S3 Fig — (DOCX) [file pone.0257606.s003.docx]
