# Supplementary material for: Somatostatin analog therapy effectiveness on the progression of polycystic kidney and liver disease: A systematic review and meta-analysis of randomized clinical trials
Source: PLoS One. 2021 Sep 24;16(9):e0257606. doi: 10.1371/journal.pone.0257606 (PMC8462725; doi:10.1371/journal.pone.0257606)
Supplement: S2 Table — (DOCX) [file pone.0257606.s005.docx]

**(S2 Table) Search strategy**

**Embase <1988 to 2018 Week 21>**

| **Search history sorted by search number ascending** | | | |  |  |  |
| --- | --- | --- | --- | --- | --- | --- |
| **#** | **Searches** | **Results** | **Type** |  |  |  |
|  | | | | | | |
| **1** | **exp somatostatin derivative/** | **44494** | **Advanced** |  |  |  |
| **2** | **kidney polycystic disease/** | **13439** | **Advanced** |  |  |  |
| **3** | **liver polycystic disease/** | **1171** | **Advanced** |  |  |  |
| **4** | **1 and (2 or 3)** | **258** | **Advanced** |  |  |  |
| **5** | **exp case control study/ or exp case study/ or exp clinical trial/ or exp intervention study/ or exp major clinical study/ or exp prospective study/ or exp retrospective study/** | **4088017** | **Advanced** |  |  |  |
| **6** | **"types of study"/ or exp comparative study/ or exp controlled study/ or exp observational study/** | **6729570** | **Advanced** |  |  |  |
| **7** | **follow up/** | **1251771** | **Advanced** |  |  |  |
| **8** | **4 and (5 or 6 or 7 or meta-analysis/ or systematic review/)** | **110** | **Advanced** |  |  |  |
| **9** | **limit 8 to human** | **101** |  |  |  |  |

**Ovid MEDLINE(R) 1946 to Present and Epub Ahead of Print, In-Process & Other Non-Indexed Citations and Ovid MEDLINE(R) Daily**

| **Search history sorted by search number ascending** | | | |  |  |  |
| --- | --- | --- | --- | --- | --- | --- |
| **#** | **Searches** | **Results** | **Type** |  |  |  |
|  | | | | | | |
| **1** | **exp somatostatin/** | **18563** | **Advanced** |  |  |  |
| **2** | **(somatostatin* or octreotide or angiopeptin or paseriotide or pentretide).mp. [mp=title, abstract, original title, name of substance word, subject heading word, keyword heading word, protocol supplementary concept word, rare disease supplementary concept word, unique identifier, synonyms]** | **36795** | **Advanced** |  |  |  |
| **3** | **1 or 2** | **36795** | **Advanced** |  |  |  |
| **4** | **exp Polycystic Kidney Diseases/** | **9017** | **Advanced** |  |  |  |
| **5** | **(polycystic adj2 (kidney or renal or hepatic or liver)).mp. [mp=title, abstract, original title, name of substance word, subject heading word, keyword heading word, protocol supplementary concept word, rare disease supplementary concept word, unique identifier, synonyms]** | **12104** | **Advanced** |  |  |  |
| **6** | **4 or 5** | **12104** | **Advanced** |  |  |  |
| **7** | **3 and 6** | **111** | **Advanced** |  |  |  |
| **8** | **limit 7 to (adaptive clinical trial or clinical trial, all or clinical trial, phase i or clinical trial, phase ii or clinical trial, phase iii or clinical trial, phase iv or clinical trial or comparative study or controlled clinical trial or equivalence trial or evaluation studies or meta analysis or multicenter study or observational study or pragmatic clinical trial or randomized controlled trial or systematic reviews)** | **31** | **Advanced** |  |  |  |
| **9** | **7 and (observational* or trial* or "meta analysis" or prospective* or retrospective* or "systematic review*").mp. [mp=title, abstract, original title, name of substance word, subject heading word, keyword heading word, protocol supplementary concept word, rare disease supplementary concept word, unique identifier, synonyms]** | **59** | **Advanced** |  |  |  |
| **10** | **8 or 9** | **62** |  |  |  |  |

**CENTRAL – 27**

**Scopus**

**( ( TITLE-ABS-KEY ( ( somatostatin*  OR  octreotide  OR  angiopeptin  OR  paseriotide  OR  pentretide ) )  AND  TITLE-ABS-KEY ( ( polycystic  W/3  ( renal  OR  kidney  OR  liver  OR  hepatic ) ) ) ) )  AND  ( volume*  OR  enlarg*  OR  hepatomegal* ) 187**

**ACTUALIZACIÓN <January 2020 to November 2020>**

| **DATABASE** | Before deduplication | After deduplication |
| --- | --- | --- |
| **Scopus** | **47** | **38** |
| **Embase** | **21** | **7** |
| **Medline** | **17** | **17** |

Total= 62
All searches were run on November 4, 2020.

**SCOPUS**

( ( TITLE-ABS-KEY ( ( somatostatin* OR octreotide OR angiopeptin OR paseriotide OR pentretide ) ) AND TITLE-ABS-KEY ( ( polycystic W/3 ( renal OR kidney OR liver OR hepatic ) ) ) ) ) AND ( volume* OR enlarg* OR hepatomegal* ) AND ( LIMIT-TO ( PUBYEAR , 2021 ) OR LIMIT-TO ( PUBYEAR , 2020 ) OR LIMIT-TO ( PUBYEAR , 2019 ) OR LIMIT-TO ( PUBYEAR , 2018 ) )

47 document results

|  | **Embase**1988 to 2020 Week 44 |  |
| --- | --- | --- |
| 1 | exp somatostatin derivative/ | 49681 |
| 2 | kidney polycystic disease/ | 15863 |
| 3 | liver polycystic disease/ | 1386 |
| 4 | 1 and (2 or 3) | 320 |
| 5 | exp case control study/ or exp case study/ or exp clinical trial/ or exp intervention study/ or exp major clinical study/ or exp prospective study/ or exp retrospective study/ | 5269161 |
| 6 | "types of study"/ or exp comparative study/ or exp controlled study/ or exp observational study/ | 8689379 |
| 7 | follow up/ | 1583359 |
| 8 | 4 and (5 or 6 or 7 or meta-analysis/ or systematic review/) | 132 |
| 9 | limit 8 to human | 120 |
| 10 | limit 9 to yr="2018 -Current" | 21 |

| \| **Ovid MEDLINE(R) 1946 to Present and Epub Ahead of Print, In-Process & Other Non-Indexed Citations and Ovid MEDLINE(R) Daily** \| \| --- \| | | | |
| --- | --- | --- | --- | --- |
| 1 | exp somatostatin/ | 19195 | Advanced |
| 2 | (somatostatin* or octreotide or angiopeptin or paseriotide or pentretide).mp. | 39329 | Advanced |
| 3 | 1 or 2 | 39329 | Advanced |
| 4 | exp Polycystic Kidney Diseases/ | 9744 | Advanced |
| 5 | (polycystic adj2 (kidney or renal or hepatic or liver)).mp. | 13376 | Advanced |
| 6 | 4 or 5 | 13376 | Advanced |
| 7 | 3 and 6 | 138 | Advanced |
| 8 | limit 7 to (adaptive clinical trial or clinical trial, all or clinical trial, phase i or clinical trial, phase ii or clinical trial, phase iii or clinical trial, phase iv or comparative study or controlled clinical trial or equivalence trial or evaluation study or meta analysis or multicenter study or observational study or pragmatic clinical trial or randomized controlled trial or "systematic review") | 36 | Advanced |
| 9 | 7 and (observational* or trial* or "meta analysis" or prospective* or retrospective* or "systematic review*").mp. | 75 | Advanced |
| 10 | 8 or 9 | 77 | Advanced |
| 11 | limit 10 to yr="2018 -Current" | 17 | Advanced |
