# Supplementary material for: Somatostatin analog therapy effectiveness on the progression of polycystic kidney and liver disease: A systematic review and meta-analysis of randomized clinical trials
Source: PLoS One. 2021 Sep 24;16(9):e0257606. doi: 10.1371/journal.pone.0257606 (PMC8462725; doi:10.1371/journal.pone.0257606)
Supplement: S3 Table — (DOCX) [file pone.0257606.s006.docx]

**(S3 Table) List of excluded studies**

1. Wijnands TFM, Gevers TJG, Lantinga MA, Te Morsche RH, Schultze Kool LJ, Drenth JPH. Pasireotide does not improve efficacy of aspiration sclerotherapy in patients with large hepatic cysts, a randomized controlled trial. European Radiology. 2018;28(6):2682-2689.

2. van Aerts RMM, van de Laarschot LFM, Banales JM, Drenth JPH. Clinical management of polycystic liver disease. Journal of Hepatology. 2018;68(4):827-837.

3. Van Aerts R, Kolkman M, Nevens F, Kievit W, Drenth JPH. The effect of re-exposure to somatostatin analogues in patients with polycystic liver disease. Journal of Hepatology. 2018;68 (Supplement 1):S624.

4. Lorenzo Pisarello M, Masyuk TV, Gradilone SA, et al. Combination of a Histone Deacetylase 6 Inhibitor and a Somatostatin Receptor Agonist Synergistically Reduces Hepatorenal Cystogenesis in an Animal Model of Polycystic Liver Disease. American Journal of Pathology. 2018;188(4):981-994.

5. Wu XY, Bai XY. Advances in diagnosis and treatment of autosomal dominant polycystic kidney disease. Medical Journal of Chinese People's Liberation Army. 2017;42(11):1020-1024.

6. Wong MY, McCaughan GW, Strasser SI. An update on the pathophysiology and management of polycystic liver disease. Expert Review of Gastroenterology and Hepatology. 2017;11(6):569-581.

7. Temmerman F, Chen F, Libbrecht L, et al. Everolimus halts hepatic cystogenesis in a rodent model of polycystic-liver-disease. World Journal of Gastroenterology. 2017;23(30):5499-5507.

8. Spaho L, Fasullo M, Zacharias I. A rare case of primary hepatic neuroendocrine tumor and its non-surgical management. American Journal of Gastroenterology. 2017;112 (Supplement 1):S1272.

9. Sharma K, Caroli A, Van Quach L, et al. Kidney volume measurement methods for clinical studies on autosomal dominant polycystic kidney disease. PLoS ONE. 2017;12 (5):e0178488.

10. Santos-Laso A, Izquierdo-Sánchez L, Lee-Law PY, et al. New Advances in Polycystic Liver Diseases. Seminars in Liver Disease. 2017;37(1):45-55.

11. Reddy BV, Chapman AB. The spectrum of autosomal dominant polycystic kidney disease in children and adolescents. Pediatric Nephrology. 2017;32(1):31-42.

12. Potts JW, Mousa SA. Recent advances in management of autosomal-dominant polycystic kidney disease. American Journal of Health-System Pharmacy. 2017;74(23):1959-1968.

13. Perugorria MJ, Labiano I, Esparza-Baquer A, et al. Bile Acids in Polycystic Liver Diseases: Triggers of Disease Progression and Potential Solution for Treatment. Digestive Diseases. 2017;35(3):275-281.

14. Mikolajczyk AE, Te HS, Chapman AB. Gastrointestinal Manifestations of Autosomal-Dominant Polycystic Kidney Disease. Clinical Gastroenterology and Hepatology. 2017;15(1):17-24.

15. Masyuk TV, Masyuk AI, Lorenzo Pisarello M, et al. TGR5 contributes to hepatic cystogenesis in rodents with polycystic liver diseases through cyclic adenosine monophosphate/Galphas signaling. Hepatology. 2017;66(4):1197-1218.

16. Masyuk TV, Masyuk AI, LaRusso NF. Therapeutic Targets in Polycystic Liver Disease. Current Drug Targets. 2017;18(8):950-957.

17. Lantinga MA, D'Agnolo HM, Casteleijn NF, et al. Hepatic Cyst Infection During Use of the Somatostatin Analog Lanreotide in Autosomal Dominant Polycystic Kidney Disease: An Interim Analysis of the Randomized Open-Label Multicenter DIPAK-1 Study. Drug Safety. 2017;40(2):153-167.

18. Lanktree MB, Chapman AB. New treatment paradigms for ADPKD: Moving towards precision medicine. Nature Reviews Nephrology. 2017;13(12):750-768.

19. Kugita M, Nishii K, Yamaguchi T, et al. Beneficial effect of combined treatment with octreotide and pasireotide in PCK rats, an orthologous model of human autosomal recessive polycystic kidney disease. PLoS ONE. 2017;12(5):e0177934.

20. Kawaratani H, Fukui H, Yoshiji H. Treatment for cirrhotic ascites. Hepatology Research. 2017;47(2):166-177.

21. Iliuta IA, Kitchlu A, Pei Y. Methodological issues in clinical trials of polycystic kidney disease: a focused review. Journal of Nephrology. 2017;30(3):363-371.

22. Chen CH, Weiss RH. GHetting to know ADPKD proliferative signaling, STAT. Kidney International. 2017;91(3):524-526.

23. Butscher A, Phan O, Bonny O. Extra-renal manifestations of the autosomal dominant polycystic kidney disease. Revue Medicale Suisse. 2017;13(551):450-456.

24. Yuajit C, Chatsudthipong V. Nutraceutical for autosomal dominant polycystic kidney disease therapy. Journal of the Medical Association of Thailand. 2016;99:S97-S103.

25. Xue C, Zhou CC, Wu M, Mei CL. The Clinical Manifestation and Management of Autosomal Dominant Polycystic Kidney Disease in China. Kidney Diseases. 2016;2(3):111-119.

26. Woo YM, Ko JY, Lee EJ. Validation of effective therapeutic targets for ADPKD using animal models. Advances in Experimental Medicine and Biology. Vol 933: Springer New York LLC; 2016:71-84.

27. Wijnands TFM, Gevers TJG, Lantinga MA, Schultze Kool LJ, Drenth JPH. The effect of pasireotide in cyst reduction of aspiration sclerotherapy in patients with large symptomatic hepatic cysts, a randomized controlled trial. United european gastroenterology journal. 2016;Conference: 24th united european gastroenterology week, UEG. 2016. Austria 4(5 Supplement 1):A54.

28. Sun L, Yu CY, Vienna Mackey L, Coy DH. Lanreotide and its potential applications in polycystic kidney and liver diseases. Current Topics in Medicinal Chemistry. 2016;16(2):133-140.

29. Strutz F. Autosomal polycystic kidney disease. Deutsche Medizinische Wochenschrift. 2016;141(20):1463-1466.

30. Simms RJ. Autosomal dominant polycystic kidney disease. BMJ (Online). 2016;352:i679.

31. Rysz J, Gluba-Brzozka A, Franczyk B, Banach M, Bartnicki P. Combination drug versus monotherapy for the treatment of autosomal dominant polycystic kidney disease. Expert Opinion on Pharmacotherapy. 2016;17(15):2049-2056.

32. Rangan GK, Alexander SI, Campbell KL, et al. KHA-CARI guideline recommendations for the diagnosis and management of autosomal dominant polycystic kidney disease. Nephrology. 2016;21(8):705-716.

33. Perico N, Cortinovis M, Remuzzi G. [Treatment of Autosomal Dominant Polycystic Kidney Disease (ADPKD): Somatostatin analogues and mTOR inhibitors]. Giornale Italiano di Nefrologia. 2016;33(5):gin/33.35.19.

34. Mossaad A. Large hemorrhagic liver cyst causing bile duct obstruction. American Journal of Gastroenterology. 2016;111 (Supplement 1):S926-S927.

35. Messchendorp AL, Spithoven EM, Meijer E, et al. Somatostatin is not associated with disease severity or rate of disease progression in patients with autosomal dominant polycystic kidney disease. Nephrology Dialysis Transplantation. 2016;1):i362-i363.

36. Larusso NF, Masyuk TV, Hogan MC. Polycystic Liver Disease: The Benefits of Targeting cAMP. Clinical Gastroenterology and Hepatology. 2016;14(7):1031-1034.

37. Kim H, Hwang YH. Clinical trials and a view toward the future of ADPKD. Advances in Experimental Medicine and Biology. Vol 933: Springer New York LLC; 2016:105-121.

38. Khan S, Dennison A, Garcea G. Medical therapy for polycystic liver disease. Annals of the Royal College of Surgeons of England. 2016;98(1):18-23.

39. Jiang L, Fang P, Weemhoff JL, Apte U, Pritchard MT. Evidence for a "Pathogenic Triumvirate" in Congenital Hepatic Fibrosis in Autosomal Recessive Polycystic Kidney Disease. BioMed Research International. 2016;2016 4918798.

40. Hogan MC, Masyuk TV, Vaughan L, et al. Randomized, placebo controlled double blind clinical trial of the pan-somatostatin agonist pasireotide LAR for patients with ADPKD or ADPLD with Severe Liver Involvement. Hepatology. 2016;64 (1 Supplement 1):177A-178A.

41. Hamamoto A, Yamato S, Katoh Y, et al. Modulation of primary cilia length by melanin-concentrating hormone receptor 1. Cellular Signalling. 2016;28(6):572-584.

42. Hama T, Park F. Heterotrimeric G protein signaling in polycystic kidney disease. Physiological Genomics. 2016;48(7):429-445.

43. Gevers TJ, Nevens F, Torres VE, Hogan MC, Drenth JP. Alkaline phosphatase predicts response in polycystic liver disease during somatostatin analogue therapy: a pooled analysis. Liver International. 2016;36(4):595-602.

44. Chebib FT, Jung Y, Heyer CM, et al. Effect of genotype on the severity and volume progression of polycystic liver disease in autosomal dominant polycystic kidney disease. Nephrology Dialysis Transplantation. 2016;31(6):952-960.

45. Casteleijn NF, Messchendorp AL, Spithoven EM, et al. The DIPAK 1 study: Baseline characteristics and short-term treatment effects of lanreotide versus standard care in patients with later stage adpkd. Nephrology Dialysis Transplantation. 2016;31(21):i90-91.

46. Zeybek C, Orman H, Gök F. A general overview to the treatment of cystic renal deiseases and innovations in the treatment: Review. Turkiye Klinikleri Pediatri. 2015;24(2):51-59.

47. Yu ASL, El-Ters M, Winklhofer FT. Clinical Trials in Autosomal Dominant Polycystic Kidney Disease. Codon Publications Chapter. 2015;6:11.

48. Wijnands TF, Gevers TJ, Kool LJ, Drenth JP. Aspiration sclerotherapy combined with pasireotide to improve reduction of large symptomatic hepatic cysts (SCLEROCYST): study protocol for a randomized controlled trial. Trials [Electronic Resource]. 2015;16:82.

49. Tseng J, Orloff SL. Management of symptomatic polycystic liver disease with hepatic resection. JAMA Surgery. 2015;150(1):81-82.

50. Temmerman F, Ho TA, Vanslembrouck R, et al. Lanreotide Reduces Liver Volume, But Might Not Improve Muscle Wasting or Weight Loss, in Patients With Symptomatic Polycystic Liver Disease. Clinical Gastroenterology & Hepatology. 2015;13(13):2353-2359.e2351.

51. Silverman J, Desai C, Lerma EV. Autosomal dominant polycystic kidney disease. Disease-a-Month. 2015;61(10):442-447.

52. Savige J, Mallett A, Tunnicliffe DJ, Rangan GK. KHA-CARI Autosomal Dominant Polycystic Kidney Disease Guideline: Management of Polycystic Liver Disease. Seminars in Nephrology. 2015;35(6):618-622.e615.

53. Santoro D, Pellicano V, Visconti L, Trifiro G, Buemi M, Cernaro V. An overview of experimental and early investigational therapies for the treatment of polycystic kidney disease. Expert Opinion on Investigational Drugs. 2015;24(9):1199-1218.

54. Sampaziotis F, De Brito MC, Madrigal P, et al. Cholangiocytes derived from human induced pluripotent stem cells for disease modeling and drug validation. Nature Biotechnology. 2015;33(8):845-852.

55. Pisani A, Ruggenenti P, Remuzzi G, Riccio E, Sabbatini M. Long term effects of octreotide treatment in ADPKD patients with polycystic liver disease (a sub-group analysis of the ALADIN trial). Nephrology Dialysis Transplantation Conference: 52nd ERA EDTA Congress London United Kingdom Conference Start. 2015;30:iii62.

56. Nota CLMA, Molenaar IQ, Borel Rinkes IHM, Hagendoorn J. Robot-assisted laparoscopic fenestration of giant hepatic cysts. Surgical Laparoscopy, Endoscopy and Percutaneous Techniques. 2015;25(5):e163-e165.

57. Noel N, Rieu P. [Pathophysiology, epidemiology, clinical presentation, diagnosis and treatment options for autosomal dominant polycystic kidney disease]. Nephrologie et Therapeutique. 2015;11(4):213-225.

58. Neijenhuis MK, Gevers TJ, Nevens F, et al. Somatostatin analogues improve health-related quality of life in polycystic liver disease: a pooled analysis of two randomised, placebo-controlled trials. Alimentary Pharmacology & Therapeutics. 2015;42(5):591-598.

59. Neijenhuis MK, Gevers T. Update in management of polycystic liver disease. Verdauungskrankheiten. 2015;33(1):10-17.

60. Mallett A, Lee VW, Mai J, Lopez-Vargas P, Rangan GK. KHA-CARI Autosomal Dominant Polycystic Kidney Disease Guideline: Pharmacological Management. Seminars in Nephrology. 2015;35(6):582-589.e517.

61. Lazaridis KN, Larusso NF. The cholangiopathies. Mayo Clinic Proceedings. 2015;90(6):791-800.

62. LaRiviere WB, Irazabal MV, Torres VE. Novel therapeutic approaches to autosomal dominant polycystic kidney disease. Translational Research: The Journal Of Laboratory & Clinical Medicine. 2015;165(4):488-498.

63. Kazancioglu R, Gursu M. New options in the treatment of autosomal dominant polycystic kidney disease. Renal Failure. 2015;37(4):535-541.

64. Hopp K, Hommerding CJ, Wang X, Ye H, Harris PC, Torres VE. Tolvaptan plus pasireotide shows enhanced efficacy in a PKD1 model. Journal of the American Society of Nephrology. 2015;26(1):39-47.

65. Hogan MC, Masyuk T, Bergstralh E, et al. Efficacy of 4 Years of Octreotide Long-Acting Release Therapy in Patients With Severe Polycystic Liver Disease. Mayo Clinic Proceedings. 2015;90(8):1030-1037.

66. Higashihara E, Nutahara K, Okegawa T, et al. Safety study of somatostatin analogue octreotide for autosomal dominant polycystic kidney disease in Japan. Clinical & Experimental Nephrology. 2015;19(4):746-752.

67. Gevers TJ, Hol JC, Monshouwer R, Dekker HM, Wetzels JF, Drenth JP. Effect of lanreotide on polycystic liver and kidneys in autosomal dominant polycystic kidney disease: an observational trial. Liver International. 2015;35(5):1607-1614.

68. D'Agnolo HM, Gevers TJ, Riano I, et al. Efficacy of ursodeoxycholic acid as a volume reducing treatment for symptomatic polycystic liver disease: An international, multicenter, randomized controlled trial. Journal of hepatology. 2015;62(22):S848-849.

69. Chapman AB, Devuyst O, Eckardt KU, et al. Autosomal-dominant polycystic kidney disease (ADPKD): Executive summary from a Kidney Disease: Improving Global Outcomes (KDIGO) Controversies Conference. Kidney International. 2015;88(1):17-27.

70. Bolignano D, Palmer SC, Ruospo M, Zoccali C, Craig JC, Strippoli GF. Interventions for preventing the progression of autosomal dominant polycystic kidney disease. Cochrane Database of Systematic Reviews. 2015(7):CD010294.

71. Alam A. Risk factors for progression in ADPKD. Current Opinion in Nephrology and Hypertension. 2015;24(3):290-294.

72. Akoh JA. Current management of autosomal dominant polycystic kidney disease. World Journal of Nephrology. 2015;4(4):468-479.

73. Wuthrich RP, Mei C. Pharmacological management of polycystic kidney disease. Expert Opinion on Pharmacotherapy. 2014;15(8):1085-1095.

74. Wills ES, Roepman R, Drenth JPH. Polycystic liver disease: Ductal plate malformation and the primary cilium. Trends in Molecular Medicine. 2014;20(5):261-270.

75. Wijnands TFM, Neijenhuis MK, Kievit W, et al. Evaluating health-related quality of life in patients with polycystic liver disease and determining the impact of symptoms and liver volume. Liver International. 2014;34(10):1578-1583.

76. Treille S, Bailly JM, Guillaume B. Lanreotide in polycystic kidney disease: A light at the end of the tunnel? Nephrology Dialysis Transplantation. 2014;29(31):iii76.

77. Torres VE, Harris PC. Strategies targeting cAMP signaling in the treatment of polycystic kidney disease. Journal of the American Society of Nephrology. 2014;25(1):18-32.

78. Torra R. [Treatment of autosomal dominant polycystic kidney disease]. Medicina Clinica. 2014;142(2):73-79.

79. Temmerman F, Dobbels F, Ho TA, et al. Development and validation of a polycystic liver disease complaint-specific assessment (POLCA). Journal of Hepatology. 2014;61(5):1143-1150.

80. Riella C, Czarnecki PG, Steinman TI. Therapeutic advances in the treatment of polycystic kidney disease. Nephron - Clinical Practice. 2014;128:297-302.

81. Perugorria MJ, Masyuk TV, Marin JJ, et al. Polycystic liver diseases: Advanced insights into the molecular mechanisms. Nature Reviews Gastroenterology and Hepatology. 2014;11(12):750-761.

82. Neijenhuis M, Gevers TJ, Kievit W, et al. Somatostatin analogues improve health related quality of life in polycystic liver disease. United European Gastroenterology Journal. 2014;1):A450.

83. Myint TM, Rangan GK, Webster AC. Treatments to slow progression of autosomal dominant polycystic kidney disease: systematic review and meta-analysis of randomized trials. Nephrology. 2014;19(4):217-226.

84. Meijer E, Drenth JP, d'Agnolo H, et al. Rationale and design of the DIPAK 1 study: a randomized controlled clinical trial assessing the efficacy of lanreotide to Halt disease progression in autosomal dominant polycystic kidney disease. American Journal of Kidney Diseases. 2014;63(3):446-455.

85. Mahnensmith RL. Novel treatments of autosomal dominant polycystic kidney disease. Clinical Journal of the American Society of Nephrology. 2014;9(5):831-836.

86. Luciano RL, Dahl NK. Extra-renal manifestations of autosomal dominant polycystic kidney disease (ADPKD): Considerations for routine screening and management. Nephrology Dialysis Transplantation. 2014;29(2):247-254.

87. Liou IW. Management of end-stage liver disease. Medical Clinics of North America. 2014;98(1):119-152.

88. Kanaan N, Devuyst O, Pirson Y. Renal transplantation in autosomal dominant polycystic kidney disease. Nature Reviews Nephrology. 2014;10(8):455-465.

89. Johnson DK, Panchili S, Kolasseri S, Mavali RT. Polycystic liver disease presenting as pruritus. Annals of Gastroenterology. 2014;27(1):76-78.

90. Hartung EA, Guay-Woodford LM. Autosomal recessive polycystic kidney disease: A hepatorenal fibrocystic disorder with pleiotropic effects. Pediatrics. 2014;134(3):e833-e845.

91. Gall ECL, Le Meur Y. Polycystic kidney disease: Kidney volume-a crystal ball for ADPKD prognosis? Nature Reviews Nephrology. 2014;10(9):485-486.

92. Cornec-Le Gall E, Le Meur Y. [Autosomal dominant polycystic kidney disease: is the treatment for tomorrow?]. Nephrologie et Therapeutique. 2014;10(6):433-440.

93. Cnossen WR, Drenth JPH. Polycystic liver disease: An overview of pathogenesis, clinical manifestations and management. Orphanet Journal of Rare Diseases. 2014;9 (1):69.

94. Bolignano D, Palmer S, Ruospo M, Zoccali C, Craig J, Strippoli G. Interventions for retarding the progression of autosomal dominant polycystic kidney disease (ADPKD): A systematic reviewand meta-analysis. Nephrology Dialysis Transplantation. 2014;3):iii5.

95. Bertino G, Ardiri A, Demma S, et al. Rare benign tumors of the liver: Still rare? Journal of Gastrointestinal Cancer. 2014;45(2):202-217.

96. Ampuero J, Bañales JM, Soriano G, et al. Polycystic liver in the adult (PLA) in Spain: Analysis of a structured survey analysing the experience and attitude of Spanish gastroenterologists. Revista Espanola de Enfermedades Digestivas. 2014;106(4):263-275.

97. Temmerman FJ, Ho TA, Vanslembrouck R, et al. Open label, phase-II clinical study, to evaluate the efficacy of lanreotide 90mg in symptomatic polycystic liver disease, including dose escalation at month 6 in nonresponders. Hepatology. 2013;1):299A.

98. Temmerman F, Gevers T, Ho TA, et al. Safety and efficacy of different lanreotide doses in the treatment of polycystic liver disease: pooled analysis of individual patient data. Alimentary pharmacology & therapeutics. 2013;38(4):397-406.

99. Shams R. The mass, the rash, the murmur. American Journal of Gastroenterology. 2013;1):S112-S113.

100. Serra AL, Petzold K. Current therapy studies on the indications for cystic kidney diseases. Nephrologe. 2013;8(5):396-405.

101. Qian Q, Wang HY. ALADIN: Wish granted in inherited polycystic kidney disease? The Lancet. 2013;382(9903):1469-1471.

102. Pan J, Seeger-Nukpezah T, Golemis EA. The role of the cilium in normal and abnormal cell cycles: Emphasis on renal cystic pathologies. Cellular and Molecular Life Sciences. 2013;70(11):1849-1874.

103. Nunes JM, Rodrigues E, Vinha E, et al. Acromegaly in a patient with multiple endocrine disorders. Endocrine Reviews Conference: 95th Annual Meeting and Expo of the Endocrine Society, ENDO. 2013;34(3 SUPPL. 1).

104. Mochizuki T, Tsuchiya K, Nitta K. Autosomal dominant polycystic kidney disease: Recent advances in pathogenesis and potential therapies. Clinical and Experimental Nephrology. 2013;17(3):317-326.

105. Meijer E, Drenth JPH, De Fijter JW, et al. Lanreotide protects the kidney function in polycystic kidney disease? Nederlands Tijdschrift voor Geneeskunde. 2013;157(14):A5867.

106. Masyuk TV, Radtke BN, Stroope AJ, et al. Pasireotide is more effective than octreotide in reducing hepatorenal cystogenesis in rodents with polycystic kidney and liver diseases. Hepatology. 2013;58(1):409-421.

107. Liebau MC, Serra AL. Looking at the (w)hole: magnet resonance imaging in polycystic kidney disease. Pediatric Nephrology. 2013;28(9):1771-1783.

108. Jardine MJ, Liyanage T, Buxton E, Perkovic V. MTOR inhibition in autosomal-dominant polycystic kidney disease (ADPKD): The question remains open. Nephrology Dialysis Transplantation. 2013;28(2):242-244.

109. Irazabal MV, Torres VE. Experimental therapies and ongoing clinical trials to slow down progression of ADPKD. Current Hypertension Reviews. 2013;9(1):44-59.

110. Gevers TJ, Inthout J, Caroli A, et al. Young women with polycystic liver disease respond best to somatostatin analogues: a pooled analysis of individual patient data. Gastroenterology. 2013;145(2):357-365.e351-352.

111. Gevers TJ, Drenth JP. Diagnosis and management of polycystic liver disease. Nature Reviews Gastroenterology & Hepatology. 2013;10(2):101-108.

112. Everson GT, Helmke SM. Somatostatin, estrogen, and polycystic liver disease. Gastroenterology. 2013;145(2):279-282.

113. Devuyst O, Torres VE. Osmoregulation, vasopressin, and cAMP signaling in autosomal dominant polycystic kidney disease. Current Opinion in Nephrology and Hypertension. 2013;22(4):459-470.

114. Czarnecki PG, Steinman TI. Polycystic kidney disease: New horizons and therapeutic frontiers. Minerva Urologica e Nefrologica. 2013;65(1):61-68.

115. Chrispijn M, Gevers TJG, Hol JC, Monshouwer R, Dekker HM, Drenth JPH. Everolimus does not further reduce polycystic liver volume when added to long acting octreotide: Results from a randomized controlled trial in polycystic liver disease patients. Journal of Hepatology. 2013;1):S557.

116. Chrispijn M, Gevers TJ, Hol JC, Monshouwer R, Dekker HM, Drenth JP. Everolimus does not further reduce polycystic liver volume when added to long acting octreotide: results from a randomized controlled trial. Journal of Hepatology. 2013;59(1):153-117. Chang MY, Ong ACM. New treatments for autosomal dominant polycystic kidney disease. British Journal of Clinical Pharmacology. 2013;76(4):524-535.

118. Büscher R, Büscher AK, Weber S, et al. Clinical manifestations of autosomal recessive polycystic kidney disease (ARPKD): kidney-related and non-kidney-related phenotypes. Pediatric Nephrology. 2013;29(10):1915-1925.

119. Blanco G, Wallace DP. Novel role of ouabain as a cystogenic factor in autosomal dominant polycystic kidney disease. American Journal of Physiology - Renal Physiology. 2013;305(6):F797-F812.

120. Bakoyiannis A, Delis S, Triantopoulou C, Dervenis C. Rare cystic liver lesions: A diagnostic and managing challenge. World Journal of Gastroenterology. 2013;19(43):7603-7619.

121. Allison SJ. Trial of a long-acting somatostatin analogue for autosomal dominant polycystic kidney disease. Nature Reviews Nephrology. 2013;9(10):553.

122. Aguiari G, Catizone L, Del Senno L. Multidrug therapy for polycystic kidney disease: A review and perspective. American Journal of Nephrology. 2013;37(2):175-182.

123. Abu-Wasel B, Walsh C, Keough V, Molinari M. Pathophysiology, epidemiology, classification and treatment options for polycystic liver diseases. World Journal of Gastroenterology. 2013;19(35):5775-5786.

124. Syro LV, Sundsbak JL, Scheithauer BW, et al. Somatotroph pituitary adenoma with acromegaly and autosomal dominant polycystic kidney disease: SSTR5 polymorphism and PKD1 mutation. Pituitary. 2012;15(3):342-349.

125. Steinman TI. Polycystic kidney disease: a 2011 update. Current Opinion in Nephrology & Hypertension. 2012;21(2):189-194.

126. Spirli C, Morell CM, Locatelli L, et al. Cyclic AMP/PKA-dependent paradoxical activation of Raf/MEK/ERK signaling in polycystin-2 defective mice treated with sorafenib. Hepatology. 2012;56(6):2363-2374.

127. Ruggenenti P, Gaspari F, Cannata A, et al. Measuring and estimating GFR and treatment effect in ADPKD patients: Results and implications of a longitudinal cohort study. PLoS ONE. 2012;7 (2):e32533.

128. Macutkiewicz C, Plastow R, Chrispijn M, et al. Complications arising in simple and polycystic liver cysts. World Journal of Hepatology. 2012;4(12):406-411.

129. Hogan MC, Masyuk TV, Page L, et al. Somatostatin analog therapy for severe polycystic liver disease: results after 2 years. Nephrology Dialysis Transplantation. 2012;27(9):3532-3539.

130. Ho TA, Decleire PY, Lafontaine JJ, Pirson Y. Night blindness in a haemodialysed ADPKD patient receiving octreotide. Clinical Kidney Journal. 2012;5(5):474-475.

131. Gevers TJ, J.I TH, Caroli A, et al. Young female patients with polycystic liver disease benefit the most from somatostatin analogue therapy: An individual patient data meta-analysis. Hepatology. 2012;1):828A-829A.

132. Gevers TJ, Chrispijn M, Wetzels JF, Drenth JP. Rationale and design of the RESOLVE trial: lanreotide as a volume reducing treatment for polycystic livers in patients with autosomal dominant polycystic kidney disease. BMC Nephrology. 2012;13:17.

133. Chrispijn M, Nevens F, Gevers TJ, et al. The long-term outcome of patients with polycystic liver disease treated with lanreotide. Alimentary Pharmacology & Therapeutics. 2012;35(2):266-274.

134. Chang MY, Ong AC. Mechanism-based therapeutics for autosomal dominant polycystic kidney disease: recent progress and future prospects. Nephron. 2012;120(1):c25-34; discussion c35.

135. Chandok N. Polycystic liver disease: A clinical review. Annals of Hepatology. 2012;11(6):819-826.

136. Bergmann C. Educational paper; ciliopathies. European Journal of Pediatrics. 2012;171(9):1285-1300.

137. Wallace DP. Cyclic AMP-mediated cyst expansion. Biochimica et Biophysica Acta - Molecular Basis of Disease. 2011;1812(10):1291-1300.

138. van Gulick JJM, Gevers TJG, van Keimpema L, Drenth JPH. Hepatic and renal manifestations in autosomal dominant polycystic kidney disease: A dichotomy of two ends of a spectrum. Netherlands Journal of Medicine. 2011;69(9):367-371.

139. Temmerman FJ, Vanslembrouck R, Coudyzer W, et al. Total liver volume measurement by ct corrected for body surface area is an objective parameter of the extent of polycystic liver disease. Hepatology. 2011;1):667A-668A.

140. Temmerman F, Missiaen L, Bammens B, et al. Systematic review: the pathophysiology and management of polycystic liver disease. Alimentary Pharmacology & Therapeutics. 2011;34(7):702-713.

141. Takiar V, Caplan MJ. Polycystic kidney disease: Pathogenesis and potential therapies. Biochimica et Biophysica Acta - Molecular Basis of Disease. 2011;1812(10):1337-1343.

142. Sun Y, Zhou H, Yang BX. Drug discovery for polycystic kidney disease. Acta Pharmacologica Sinica. 2011;32(6):805-816.

143. Peces R, Cuesta-López E, Peces C, Pérez-Dueñas V, Vega-Cabrera C, Selgas R. Octreotide reduces hepatic, renal and breast cystic volume in autosomal-dominant polycystic kidney disease. International Urology and Nephrology. 2011;43(2):565-569.

144. Maxwell AP. Genetic renal abnormalities. Medicine. 2011;39(6):343-349.

145. Martín RS, Fraga AR, Guillermo F, et al. Specific treatments for autosomal dominant polycystic Kidney disease. A complerx biology and a long duration disease. Revista de Nefrologia, Dialisis y Trasplante. 2011;31(2):77-85.

146. Lee K, Battini L, Gusella GL. Cilium, centrosome and cell cycle regulation in polycystic kidney disease. Biochimica et Biophysica Acta - Molecular Basis of Disease. 2011;1812(10):1263-1271.

147. Horie S. [Autosomal dominant polycystic kidney disease]. Nippon Jinzo Gakkai Shi Japanese Journal of Nephrology. 2011;53(1):6-9.

148. Gevers TJ, Drenth JP. Somatostatin analogues for treatment of polycystic liver disease. Current Opinion in Gastroenterology. 2011;27(3):294-300.

149. Chrispijn M, Drenth JP. Everolimus and long acting octreotide as a volume reducing treatment of polycystic livers (ELATE): study protocol for a randomized controlled trial. Trials. 2011;12( ):246.

150. Burtey S. [Slow the pace of renal failure in autosomal dominant polycystic kidney disease: hopes and disappointments]. Presse Medicale. 2011;40(11):1059-1064.

151. Bastos AP, Onuchic LF. Molecular and cellular pathogenesis of autosomal dominant polycystic kidney disease. Brazilian Journal of Medical and Biological Research. 2011;44(7):606-617.

152. Woudenberg J, Janssen MJ, Worm I, Te Morsche RH, Chrispijn M, Drenth JPH. Human polycystic liver cyst fluid induced cholangiocyte proliferation is reversed by lanreotide in a somatostatin receptor 5 and phosphodiesterase 4D dependent mechanism. Journal of Hepatology. 2010;1):S384-S385.

153. van Keimpema L, Drenth JPH. Effect of octreotide on polycystic liver volume. Liver International. 2010;30(4):633-634.

154. Torres VE. Treatment strategies and clinical trial design in ADPKD. Advances in Chronic Kidney Disease. 2010;17(2):190-204.

155. Serra AL, Poster D, Wüthrich RP. Autosomal dominant polycystic kidney disease: Novel therapeutic options. Nephrologe. 2010;5(5):404-411.

156. Schrier RW, Levi M. Lipids and renal cystic disease. Nephrology Dialysis Transplantation. 2010;25(11):3490-3492.

157. Schrier RW. Randomized intervention studies in human polycystic kidney and liver disease. Journal of the American Society of Nephrology. 2010;21(6):891-893.

158. Qian Q. Isolated Polycystic Liver Disease. Advances in Chronic Kidney Disease. 2010;17(2):181-189.

159. Pirson Y. Extrarenal Manifestations of Autosomal Dominant Polycystic Kidney Disease. Advances in Chronic Kidney Disease. 2010;17(2):173-180.

160. Otto EA, Hurd TW, Airik R, et al. Candidate exome capture identifies mutation of SDCCAG8 as the cause of a retinal-renal ciliopathy. Nature Genetics. 2010;42(10):840-850.

161. Ong ACM, Devuyst O. Towards the integration of genetic knowledge into clinical practice. Nephron - Clinical Practice. 2010;118(1):c3-c8.

162. Melander C, Joly D, Knebelmann B. Autosomal dominant polycystic kidney disease: Light at the end of the tunnel? Nephrologie et Therapeutique. 2010;6(4):226-231.

163. Le Pane C, Alrayes A, Abuhmaid F, Brown K. Polycystic liver disease inducing right atrial dysfunction: A case report. American Journal of Gastroenterology. 2010;1):S287.

164. Janssen MJ, Waanders E, Woudenberg J, Lefeber DJ, Drenth JPH. Congenital disorders of glycosylation in hepatology: The example of polycystic liver disease. Journal of Hepatology. 2010;52(3):432-440.

165. Ireland R. Polycystic kidney disease: Promising new potential therapies for patients with autosomal dominant polycystic kidney disease. Nature Reviews Nephrology. 2010;6(8):443.

166. Halvorson CR, Bremmer MS, Jacobs SC. Polycystic kidney disease: Inheritance, pathophysiology, prognosis, and treatment. International Journal of Nephrology and Renovascular Disease. Vol 32010:69-83.

167. Drenth JP, Chrispijn M, Nagorney DM, Kamath PS, Torres VE. Medical and surgical treatment options for polycystic liver disease1. Hepatology. 2010;52(6):2223-2230.

168. Chapin HC, Caplan MJ. The cell biology of polycystic kidney disease. Journal of Cell Biology. 2010;191(4):701-710.

169. Bulté K, Holvoet A, Aerts R, Pirenne J, Nevens F. Pathogenesis and treatment of polycystic liver disease: New insights. Tijdschrift voor Geneeskunde. 2010;66(14-15):713-718.

170. Bonny O, Chehade H, Fellmann F, Qanadli SD, Barbey F. Recent insights for the polycystic kidney disease. [French]. Revue Medicale Suisse. 2010;6(238):454-459.

171. Belibi FA, Edelstein CL. Novel targets for the treatment of autosomal dominant polycystic kidney disease. Expert Opinion on Investigational Drugs. 2010;19(3):315-328.

172. Wuthrich RP, Serra AL, Kistler AD. Autosomal dominant polycystic kidney disease: new treatment options and how to test their efficacy. Kidney & Blood Pressure Research. 2009;32(5):380-387.

173. Van Keimpema L, Höckerstedt K. Treatment of polycystic liver disease. British Journal of Surgery. 2009;96(12):1379-1380.

174. Torres VE. Type II calcimimetics and polycystic kidney disease: Unanswered questions. Journal of the American Society of Nephrology. 2009;20(7):1421-1425.

175. Patel V, Chowdhury R, Igarashi P. Advances in the pathogenesis and treatment of polycystic kidney disease. Current Opinion in Nephrology & Hypertension. 2009;18(2):99-106.

176. Meijer E, De Jong PE, Van Der Jagt EJ, Peters DJ, Breuning MH, Gansevoort RT. Hereditary cystinuria: New insights and possible treatments. Nederlands Tijdschrift voor Geneeskunde. 2009;153(20):968-974.

177. Masyuk T, Masyuk A, LaRusso N. Cholangiociliopathies: Genetics, molecular mechanisms and potential therapies. Current Opinion in Gastroenterology. 2009;25(3):265-271.

178. Hogan M, Masyuk TV, Torres V, King BF, Kim B, LaRusso NF. OctreotideLAR inhibits hepatorenal cystogenesis in the human polycystic liver diseases. Hepatology (baltimore, md). 2009;50(4 (Suppl.)):328A.

179. Harris PC, Torres VE. Polycystic kidney disease. Annual Review of Medicine. Vol 602009:321-337.

180. Harris PC. 2008 Homer W. Smith award: Insights into the pathogenesis of polycystic kidney disease from gene discovery. Journal of the American Society of Nephrology. 2009;20(6):1188-1198.

181. Gao JS, Yang BX. Progress in treatment of autosomal dominant polycystic kidney diseases. Chinese Pharmacological Bulletin. 2009;25(2):141-144.

182. Wolyniec W, Jankowska MM, Rutkowski B. [Modern treatment of autosomal dominant polycystic kidney disease]. Polski Merkuriusz Lekarski. 2008;25(148):374-379.

183. Van Keimpema L, De Man RA, Drenth JPH. Somatostatin analogues reduce liver volume in polycystic liver disease. Gut. 2008;57(9):1338-1339.

184. Rizk D, Chapman A. Treatment of autosomal dominant polycystic kidney disease (ADPKD): The new horizon for children with ADPKD. Pediatric Nephrology. 2008;23(7):1029-1036.

185. Meijer E, de Jong PE, Peters DJ, Gansevoort RT. Better understanding of ADPKD results in potential new treatment options: ready for the cure? Journal of Nephrology. 2008;21(2):133-138.

186. Ibraghimov-Beskrovnaya O, Bukanov N. Polycystic kidney diseases: From molecular discoveries to targeted therapeutic strategies. Cellular and Molecular Life Sciences. 2008;65(4):605-619.

187. Gross P. Polycystic kidney disease: Will it become treatable? Polskie Archiwum Medycyny Wewnetrznej. 2008;118(5):298-301.

188. Everson GT, Helmke SM, Doctor B. Advances in management of polycystic liver disease. Expert Review of Gastroenterology and Hepatology. 2008;2(4):563-576.

189. Edelstein CL. Therapeutic interventions for autosomal dominant polycystic kidney disease. Nephrology News & Issues. 2008;22(3):25-26.

190. Alper SL. Let's look at cysts from both sides now. Kidney International. 2008;74(6):699-702.

191. Torres VE, Rossetti S, Harris PC. Update on autosomal dominant polycystic kidney disease. Minerva Medica. 2007;98(6):669-691.

192. Torres VE, Harris PC, Pirson Y. Autosomal dominant polycystic kidney disease. Lancet. 2007;369(9569):1287-1301.

193. Torres VE, Harris PC. Polycystic kidney disease: Genes, proteins, animal models, disease mechanisms and therapeutic opportunities. Journal of Internal Medicine. 2007;261(1):17-31.

194. Torres VE. Treatment of Polycystic Liver Disease: One Size Does Not Fit All. American Journal of Kidney Diseases. 2007;49(6):725-728.

195. Serra AL, Wüthrich RP. Autosomal dominant polycystic kidney disease. Nephrologe. 2007;2(6):459-468.

196. Rapoport J. Autosomal dominant polycystic kidney disease: Pathophysiology and treatment. Qjm. 2007;100(1):1-9.

197. Maxwell AP. Genetic renal abnormalities. Medicine. 2007;35(7):386-392.

198. Masyuk TV, Masyuk AI, Torres VE, Harris PC, Larusso NF. Octreotide Inhibits Hepatic Cystogenesis in a Rodent Model of Polycystic Liver Disease by Reducing Cholangiocyte Adenosine 3′,5′-Cyclic Monophosphate. Gastroenterology. 2007;132(3):1104-1116.

199. Masoumi A, Reed-Gitomer B, Kelleher C, Schrier RW. Potential pharmacological interventions in polycystic kidney disease. Drugs. 2007;67(17):2495-2510.

200. Chapman AB. Autosomal dominant polycystic kidney disease: Time for a change? Journal of the American Society of Nephrology. 2007;18(5):1399-1407.

201. Walz G. Therapeutic approaches in autosomal dominant polycystic kidney disease (ADPKD): Is there light at the end of the tunnel? Nephrology Dialysis Transplantation. 2006;21(7):1752-1757.

202. Torres VE. Water for ADPKD? Probably, yes. Journal of the American Society of Nephrology. 2006;17(8):2089-2091.

203. Perrone R. Imaging progression in polycystic kidney disease. New England Journal of Medicine. 2006;354(20):2181-2183.

204. Grantham JJ. Does extended-release somatostatin slow the growth of renal cysts in autosomal- dominant polycystic kidney disease? Nature Clinical Practice Nephrology. 2006;2(2):66-67.

205. Gattone IVH. Emerging therapies for polycystic kidney disease. Current Opinion in Pharmacology. 2005;5(5 SPEC.ISS.):535-542.

206. Arnold HL, Harrison SA. New advances in evaluation and management of patients with polycystic liver disease. American Journal of Gastroenterology. 2005;100(11):2569-2582.

207. Peng T, Peng MH, Li LQ, et al. Intestinal perforation after combined liver-kidney transplantation for a case of congenital polycystic disease. World Journal of Gastroenterology. 2004;10(18):2769-2771.

208. Holzinger F, Baer HU, Krähenbühl L, Büchler MW. Solitary liver cysts and polycystic liver disease: Aspects of surgical management of congenital cystic liver disease. Swiss Surgery. 1999;5(3):136-142.

209. Andreeva, E. F. and N. D. Savenkova (2019). "Treatment of autosomal recessive and autosomal dominant polycystic kidney disease." Rossiyskiy Vestnik Perinatologii i Pediatrii 64(2): 22-29.

210. Aussilhou, B., et al. (2018). "Treatment of polycystic liver disease. Update on the management." Journal of Visceral Surgery 155(6): 471-481.

211. Barten, T. R. M., et al. (2020). "New insights into targeting hepatic cystogenesis in autosomal dominant polycystic liver and kidney disease." Expert opinion on therapeutic targets 24(6): 589-599.

212. Bergmann, C., et al. (2018). "Polycystic kidney disease." Nature Reviews Disease Primers 4(1).

213. Bernts, L. H. P., et al. (2019). "Management of portal hypertension and ascites in polycystic liver disease." Liver international : official journal of the International Association for the Study of the Liver 39(11): 2024-2033.

214. Bernts, L. H. P., et al. (2019). "Venous Stent Placement for Refractory Ascites due to Hepatic Venous Outflow Obstruction in Polycystic Liver Disease." Journal of Vascular and Interventional Radiology 30(10): 1617-1619.

215. Bilreiro, C. and I. Santiago (2021). Fibropolycystic Liver Diseases. Medical Radiology: 225-240.

216. Brosnahan, G. (2019). "Quest for the Cure: Testing the Old and New to Prevent Progression of Autosomal Dominant Polycystic Kidney Disease." Kidney Medicine 1(6): 329-331.

217. Chauveau, D. (2019). "Polycystic kidney and liver diseases." Hepato-Gastro et Oncologie Digestive 26(8): 830-838.

218. Chebib, F. T. and V. E. Torres (2018). "Recent Advances in the Management of Autosomal Dominant Polycystic Kidney Disease." Clinical journal of the American Society of Nephrology : CJASN 13(11): 1765-1776.

219. Colbert, G. B., et al. (2020). "Update and review of adult polycystic kidney disease." Disease-a-Month 66(5).

220. Cornec-Le Gall, E., et al. (2019). "Autosomal dominant polycystic kidney disease." The Lancet 393(10174): 919-935.

221. De Rechter, S., et al. (2018). "Unmet needs and challenges for follow-up and treatment of autosomal dominant polycystic kidney disease: The paediatric perspective." Clinical Kidney Journal 11: I14-I26.

222. Erokwu, B. O., et al. (2018). "Quantitative magnetic resonance imaging assessments of autosomal recessive polycystic kidney disease progression and response to therapy in an animal model." Pediatric Research 83(5): 1067-1074.

223. Fabris, L., et al. (2019). "Pathobiology of inherited biliary diseases: a roadmap to understand acquired liver diseases." Nature Reviews Gastroenterology and Hepatology 16(8): 497-511.

224. Galliani, M., et al. (2019). "[ADPKD treatment: Tolvaptan and Octreotide]." Giornale italiano di nefrologia : organo ufficiale della Societa italiana di nefrologia 36(6).

225. García-Prieto, A., et al. (2019). "Persistent pruritus as a rare and potentially serious manifestation of liver involvement in autosomal dominant polycystic kidney disease." Clinical Kidney Journal 12(3): 380-381.

226. Gimpel, C., et al. (2019). "International consensus statement on the diagnosis and management of autosomal dominant polycystic kidney disease in children and young people." Nature Reviews Nephrology 15(11): 713-726.

227. Griffiths, J., et al. (2020). "Long-acting somatostatin analogue treatments in autosomal dominant polycystic kidney disease and polycystic liver disease: a systematic review and meta-analysis." BMJ open 10(1): e032620.

228. K. Rangan, G., et al. (2020). "Current and emerging treatment options to prevent renal failure due to autosomal dominant polycystic kidney disease." Expert Opinion on Orphan Drugs 8(8): 285-302.

229. Khan, M., et al. (2018). "Adult polycystic liver disease: An unusual cause of recurrent variceal bleed." American Journal of Respiratory and Critical Care Medicine 197(MeetingAbstracts).

230. Kramers, B. J., et al. (2019). "Use of thiazide diuretics does not worsen disease progression in ADPKD." Nephrology Dialysis Transplantation 34(Supplement 1).

231. Kramers, B. J., et al. (2020). "Salt, but not protein intake, is associated with accelerated disease progression in autosomal dominant polycystic kidney disease." Kidney International 98(4): 989-998.

232. Lianne Messchendorp, A., et al. (2018). "Association of plasma somatostatin with disease severity and progression in patients with autosomal dominant polycystic kidney disease 11 Medical and Health Sciences 1103 Clinical Sciences." BMC nephrology 19(1).

233. Lin, C., et al. (2018). "The expression of somatostatin receptor 2 decreases during cyst growth in mice with polycystic kidney disease." Experimental biology and medicine (Maywood, N.J.) 243(13): 1092-1098.

234. Lorenzo Pisarello, M., et al. (2018). "Combination of a Histone Deacetylase 6 Inhibitor and a Somatostatin Receptor Agonist Synergistically Reduces Hepatorenal Cystogenesis in an Animal Model of Polycystic Liver Disease." American Journal of Pathology 188(4): 981-994.

235. Messchendorp, A. L., et al. (2020). "Somatostatin in renal physiology and autosomal dominant polycystic kidney disease." Nephrology, dialysis, transplantation : official publication of the European Dialysis and Transplant Association - European Renal Association 35(8): 1306-1316.

236. Messchendorp, A. L., et al. (2019). "Effect of a Somatostatin Analogue on the Vasopressin Pathway in Patients With ADPKD." Kidney International Reports 4(8): 1170-1174.

237. Messchendorp, A. L., et al. (2019). "Rapid Progression of Autosomal Dominant Polycystic Kidney Disease: Urinary Biomarkers as Predictors." American journal of nephrology 50(5): 375-385.

238. Messchendorp, A. L., et al. (2018). "Association of plasma somatostatin with disease severity and progression in patients with autosomal dominant polycystic kidney disease." BMC nephrology 19(1): 368.

239. Müller, R. U. and T. Benzing (2018). "Management of autosomal-dominant polycystic kidney disease - State-of-the-art." Clinical Kidney Journal 11: I2-I13.

240. Neijenhuis, M. K., et al. (2019). "Symptom relief and not cyst reduction determines treatment success in aspiration sclerotherapy of hepatic cysts." European radiology 29(6): 3062-3068.

241. Pisani, A., et al. (2018). "Metformin in autosomal dominant polycystic kidney disease: experimental hypothesis or clinical fact?" BMC nephrology 19(1): 282.

242. Rastogi, A., et al. (2019). "Autosomal dominant polycystic kidney disease: Updated perspectives." Therapeutics and Clinical Risk Management 15: 1041-1052.

243. Rudenko, T. E., et al. (2019). "Modern approaches to conservative therapy of polycystic kidney disease." Terapevticheskii Arkhiv 91(6): 116-123.

244. Smith, T. W., Jr., et al. (2019). "Preserving the organ donor pool and suprahepatic vena cava: Case series of transverse hepatectomy for polycystic liver disease." Annals of Hepatology.

245. Spinelli, L., et al. (2018). "Data on the assessment of LV mechanics by speckle tracking echocardiography in ADPKD patients." Data in brief 21: 2075-2081.

246. Spinelli, L., et al. (2019). "Left ventricular dysfunction in ADPKD and effects of octreotide-LAR: A cross-sectional and longitudinal substudy of the ALADIN trial." International journal of cardiology 275: 145-151.

247. Sussman, C. R., et al. (2020). "Modulation of polycystic kidney disease by G-protein coupled receptors and cyclic AMP signaling." Cellular Signalling 72.

248. Tang, S. and X. Xiao (2020). "Ferroptosis and kidney diseases." International Urology and Nephrology 52(3): 497-503.

249. Temmerman, F. and F. Nevens (2019). "Further Evidence That Lanreotide Reduces Liver Growth in Patients With Polycystic Liver Disease, But Not the End of the Story." Gastroenterology 157(2): 298-299.

250. Testa, F. and R. Magistroni (2020). "ADPKD current management and ongoing trials." Journal of Nephrology 33(2): 223-237.

251. Tran Cao, H. S., et al. (2019). "In Brief." Current Problems in Surgery 56(9).

252. van Aerts, R., et al. (2019). "Lanreotide reduces liver growth in autosomal dominant polycystic kidney disease: Data from a 120-week randomized clinical trial." Journal of Hepatology 70(1): e118-e119.

253. van Aerts, R. M. M., et al. (2018). "Clinical management of polycystic liver disease." Journal of Hepatology 68(4): 827-837.

254. Zhang, Z., et al. (2019). "Severe polycystic liver diseases: Hepatectomy or waiting for liver transplantation?: Two case reports." Medicine (United States) 98(49).

255. Zhang, Z. Y., et al. (2020). "Polycystic liver disease: Classification, diagnosis, treatment process, and clinical management." World Journal of Hepatology 12(3): 72-83.
