# Supplementary material for: Somatostatin analog therapy effectiveness on the progression of polycystic kidney and liver disease: A systematic review and meta-analysis of randomized clinical trials
Source: PLoS One. 2021 Sep 24;16(9):e0257606. doi: 10.1371/journal.pone.0257606 (PMC8462725; doi:10.1371/journal.pone.0257606)
Supplement: S4 Table — (DOCX) [file pone.0257606.s007.docx]

**(S4 Table) Summary of findings and confidence in the body of evidence**

| **Efficacy Outcomes by Scales** | **Effect Size (95% Ci)** | **No of Participants (Total Studies)** | **Quality of Evidence (Domains of Concern)** |
| --- | --- | --- | --- |
| Total liver volume | MD: -6.37 (95% CI, -7.90, -4.84) | 363 (6) | High |
| Total kidney volume | MD: -4.20 (95% CI, -5.88, -2.53) | 851 (9) | Moderate (Inconsistency) |
| eGFR | MD: -0.96 (95% CI, -2.38, 0.46) | 576 (6) | Low (Inconsistency, Indirectness) |
