# Supplementary material for: Somatostatin analog therapy effectiveness on the progression of polycystic kidney and liver disease: A systematic review and meta-analysis of randomized clinical trials
Source: PLoS One. 2021 Sep 24;16(9):e0257606. doi: 10.1371/journal.pone.0257606 (PMC8462725; doi:10.1371/journal.pone.0257606)
Supplement: S5 Table — (DOCX) [file pone.0257606.s008.docx]

**(S5 Table) calculation process to estimate ΔTLV (mean±SD) %**

**Van Aerts (2019)**

ΔTLV (%), Somatostatin group

Median (95% CI): -1.99 (-4.21～0.24)

→　SD = (Upper limit of 95%CI – Lower limit of 95%CI)/3.92 = (0.24-(-4.21))/3.92 = 1.135

ΔTLV (%), Control group

Median (95% CI): 3.92 (1.56～6.28)

→　SD = (Upper limit of 95%CI – Lower limit of 95%CI)/3.92 = (6.28-1.56)/3.92 = 1.204

**Pisani (2016)**

**Somatostatin group**

Corr (EXP) = [SD_E(base)_^2^ + SD_E(final)_^2^ – SD_E(change)_^2^]/[(2*SD_E(base)_*SD_E(final)_] = (501.2^2^ + 470.9^2^ - 133.2^2^)/(2*501.2*470.9) = 0.964357892

**Control group**

Corr (EXP) = [SD_E(base)_^2^ + SD_E(final)_^2^ – SD_E(change)_^2^]/[(2*SD_E(base)_*SD_E(final)_] = (470.7^2^ + 748.5^2^ – 316.8^2^)/(2*470.7*748.5) = 0.967090331

**Hogan (2016)**

**Somatostatin group**

Mean ΔTLV (ml) = 4104-4271 = -167, Mean ΔTLV (%) = -167/4271 = -3.3 (%)

We assume that the Correlation coefficient of this study is the average of Correlation coefficient of other studies.

Caroli (2010): Corr(EXP) = 0.993940905

Pisani (2016): Corr(EXP) = 0.964357892

The average of Correlation coefficient of these studies: 0.979149399

SD_E(change)_ = √[SD_E(base)_^2^+ SD_E(final)_^2^-(2*Corr*SD_E(base)_*SD_E(final)_)] = √(2373^2^ + 2265^2^ – 2*0.98*2373*2265) = 476 (ml)

SD ΔTLV (%) = 476/4271 = 11.1(%)

→　Mean ± SD of ΔTLV (ml); -167 ± 476 (ml)

Mean ± SD of ΔTLV (%); -3.3 ± 11.1 (%)

**Control group**

MeanΔTLV (ml) = 4294-4047 = 247 (ml), Mean ΔTLV (%) = 247 / 4047 = 6.1 (%)

We assume that the Correlation coefficient of this study is the average of Correlation coefficient of other studies.

Caroli (2010): Corr (EXP) = 0.984650924

Pisani (2016): Corr (EXP) = 0.967090331

The average of Correlation coefficient of these studies: 0.975870628

SD_E(change)_ = √[SD_E(base)_^2^+ SD_E(final)_^2^-(2*Corr*SD_E(base)_*SD_E(final)_)] = √(1298^2^ + 1314^2^ – 2*0.98*1298*1314) = 262 (ml)

SD ΔTLV (%) = 262/4047 = 6.5(%)

→　Mean ± SD of ΔTLV (ml); 247 ± 262 (ml)

Mean ± SD of ΔTLV (%); 6.1 ± 6.5 (%)

**Caroli (2010)**

**Somatostatin group**

We calculated mean ± SD of ΔTLV (%) based on the individual data of the 12 samples.

Mean ΔTLV (%) = -4.03

SD ΔTLV (%) = 3.33

→　Mean ± SD of ΔTLV (%); -4.03 ± 3.33 (%)

**Control group**

We calculated mean ± SD of ΔTLV (%) based on the individual data of the 12 samples.

Mean ΔTLV (%) = 1.23

SD ΔTLV (%) = 6.46

→　Mean ± SD of ΔTLV (%); 1.23 ± 6.46 (%)

**Van Keimpema (2009)**

**Somatostatin group**

SD ΔTLV (%) = (Upper limit of 95%CI – Lower limit of 95%CI) / 3.92 = (5.4-(-11.1)] / 3.92 = 4.2 (%)

Mean (95%CI); -2.9 (-11.1～5.4) →　Mean ± SD of ΔTLV (%); -2.9 ± 4.2 (%)

**Control group**

SD ΔTLV (%) = (Upper limit of 95%CI – Lower limit of 95%CI) / 3.92 = (8.4-(-5.2)] / 3.92 = 3.5 (%),

Mean (95%CI); 1.6 (-5.2～8.4) →　Mean ± SD of ΔTLV (%); 1.6 ± 3.5 (%)
