# Supplementary material for: Somatostatin analog therapy effectiveness on the progression of polycystic kidney and liver disease: A systematic review and meta-analysis of randomized clinical trials
Source: PLoS One. 2021 Sep 24;16(9):e0257606. doi: 10.1371/journal.pone.0257606 (PMC8462725; doi:10.1371/journal.pone.0257606)
Supplement: S6 Table — (DOCX) [file pone.0257606.s009.docx]

**(S6 Table) Calculation process to estimate ΔTKV (mean±SD) %**

**Perico (2019) 1 year**

ΔTKV (%), Somatostatin group, Pretreatment

Median (IQR): 5.2 (1.6～10.2)

→　SD = IQR/1.35 = (10.2-1.6)/1.35 = 6.37

ΔTKV (%), Somatostatin group, Posttreatment

Median (IQR): 8.8 (5.2～13.7)

→ SD = IQR/1.35 = (13.7-5.2)/1.35 = 6.15

**Perico (2019) 3 years**

ΔTKV (%), Somatostatin group, Posttreatment

Median (IQR): 29.9 (13.0～41.8)

→ SD = IQR/1.35 = (41.8-13.0)/1.35 = 21.33

ΔTKV (%), Control group, Posttreatment

Median (IQR): 37.1 (23.2～54.6)

→　SD = IQR/1.35 = (54.6-23.2)/1.35 = 23.26

**Meijer (2018) / 1 year**

**ΔhTKV (%), Somatostatin group**

Mean (95%CI): 4.15 (3.33～4.99)

→ SE = (Upper limit of 95%CI – Lower limit of 95%CI)/3.92 = (4.99-3.33)/3.92 = 0.42

SD = SE * √(n)= 0.42*√(153) = 5.195

**ΔhTKV (%), Control group**

Mean (95%CI): 5.56 (4.76～6.36)

→ SE = (Upper limit of 95%CI – Lower limit of 95%CI)/3.92 = (6.36-4.76)/3.92 = 0.41

SD = SE * √(n)= 0.41*√(152) = 5.055

**Caroli (2013) 1 year**

**ΔhTKV (%), Somatostatin group**

Mean (SE): 46.2 (18.2)

→ SD = SE *√(n) = 18.2*√(40) = 115.1

→　Mean ΔhTKV (%) = 46.2 / 1556.9 = 2.97 %, SD ΔhTKV (%) = 115.1206.6 / 1556.9 = 7.413.3 %

**ΔhTKV (%), Control group**

Mean (SE): 143.7 (26.0)

→ SD = SE*√(n) = 26.0*√(39) = 162.37

→　Mean ΔhTKV (%) = 143.7/2161.2 = 6.65 %, SD ΔhTKV (%) = 114.79/2161.2 = 5.31 %

**Caroli (2013) 3 year**

**ΔTKV (%), Somatostatin group**

Mean (SE): 220.1 (49.1)

→ SD = SE*√(n) = 49.1*√(40) = 310.54

→　Mean ΔTKV (%) = 220.1/1556.9 = 14.14 %, SD ΔTKV (%) = 310.54/1556.9 = 19.95 %

**ΔTKV (ml) Control group**

Mean (SE): 454.3 (80.8)

→ SD = SE*√(n) = 80.8*√(39) = 504.60

→　Mean ΔTKV (%) = 454.3/1556.9 = 21.02 %, SD ΔTKV (%) = 504.60/1556.9 = 32.41 %

**Van Keimpema (2009)**

**Somatostatin group**

ΔTKV (%)

Mean (95%CI): -1.5 (-13.2～10.3)

→

SE ΔTKV (%) = (Upper limit of 95%CI – Lower limit of 95%CI)/3.92 = [10.3-(-13.2)]/3.92 = 5.99

SD ΔTKV (%) = SE*√(n) = 5.99*√(27) = 31.12

**Control group**

ΔTKV (%)

Mean (95%CI): 3.4 (-7.1～14.0)

→　SEΔTKV (%) = (Upper limit of 95%CI – Lower limit of 95%CI)/3.92 = [14.0-(-7.1)]/3.92 = 5.38

SD ΔTKV (%) = SE*√(n) = 5.38*√(27) = 28.00
