# Supplementary material for: Somatostatin analog therapy effectiveness on the progression of polycystic kidney and liver disease: A systematic review and meta-analysis of randomized clinical trials
Source: PLoS One. 2021 Sep 24;16(9):e0257606. doi: 10.1371/journal.pone.0257606 (PMC8462725; doi:10.1371/journal.pone.0257606)
Supplement: S7 Table — (DOCX) [file pone.0257606.s010.docx]

**(S7 Table) Calculation process to estimate eGFR (mean±SD) mL/min./1.73m^2^**

**Perico (2019) 1 year**

eGFR, Somatostatin group, Pretreatment

Median (IQR): 27.9 (23.5～32.1)

→　SD = IQR/1.35 = (32,1-23.5)/1.35 = 6.37

eGFR, Somatostatin group, Posttreatment

Median (IQR): 22.5 (17.3～27.7)

→ SD = IQR/1.35 = (27.7-17.3)/1.35 = 7.70

eGFR, Control group, Pretreatment

Median (IQR): 25.8 (19.5～33.2)

→　SD = IQR/1.35 = (33.2-19.5)/1.35 = 10.15

eGFR, Control group, Posttreatment

Median (IQR): 20.2 (14.7～28.1)

→　SD = IQR/1.35 = (28.1-14.7)/1.35 = 9.93

We assume the Correlation coefficient is same between 1 year and 3 years.

The Correlation coefficient at 3 years is calculated as followings.

Corr (EXP) = [SD_E(base)_^2^ + SD_E(final)_^2^ – SD_E(change)_^2^]/[(2*SD_E(base)_*SD_E(final)_] = (6.37^2^ + 6.59^2^ – 2.00^2^)/(2*6.37*6.59) = 0.952932825Corr (CON) = [SD_C(base)_^2^ + SD_C(final)_^2^ – SD_C(change)_^2^]/[(2*SD_C(base)_*SD_C(final)_] = (10.15^2^ + 12.52^2^ – 2.52^2^)/(2*10.15*12.52) = 0.997113977

Therefore, SD(change) are calculated as followings.

Using this Correlation coefficient at 3 years, we calculated SD(change) at 1 year as followings.

SD_E(change)_ = √[SD_E(base)_^2^+ SD_E(final)_^2^-(2*Corr*SD_E(base)_*SD_E(final)_)] = √(6.37^2^ + 7.70^2^ – 2*0.953*6.37*7.70) = 2.52

SD_C(change)_ = √[SD_C(base)_^2^+ SD_C(final)_^2^-(2*Corr*SD_C(base)_*SD_C(final)_)] = √(10.15^2^ + 9.93^2^ – 2*0.997*10.15*9.93.) = 0.81

→ Mean ± SD (Somatostatin group, 1year): -6.2 ± 2.52

Mean ± SD (Placebo group, 1 year): -6.5 ± 0.81

**Perico (2019) 3 years**

eGFR, Somatostatin group, Posttreatment

Median (IQR): 14.9 (11.3～20.2)

→ SD = IQR/13.5 = (20.2-11.3)/1.35 = 6.59

eGFR, Control group, Posttreatment

Median (IQR): 15.0 (7.5～24.4)

→　SD = IQR/1.35 = (24.4-7.5)/1.35 = 12.52

ΔeGFR (ml), Somatostatin group (at 3 years)

Median (IQR): -5.2 (-6.2～-3.5)

→ SD = IQR/1.35 = [-3.5-(-6.2)]/1.35 = 2.00

ΔeGFR (ml), Placebo group (at 3 years)

Median (IQR): -4.7 (-6.6～-3.2)

→ SD = IQR/1.35 = [-3.2-(-6.6)]/1.35 = 2.52

**Meijer (2018)**

**ΔeGFR (ml), Somatostatin group**

Mean (95%CI): -3.53 (-4.00～3.07)

→ SE = (Upper limit of 95%CI – Lower limit of 95%CI)/3.92 = [-3.07-(-4.00)]/3.92 = 0.2372

SD = SE * √(n)= 0.2372*√(153) = 2.93

**ΔeGFR (%), Somatostatin group**

Mean (95%CI): -3.46 (-3.89～-3.02)

→ SE = (Upper limit of 95%CI – Lower limit of 95%CI)/3.92 = [-3.02-(-3.46)]/3.92 = 0.1122

SD = SE * √(n)=0.1122*√(153) = 1.39

**Caroli (2013) 1 year**

**Somatostatin group, baseline**

SD pretreatment eGFR = SE*√n = 3.93*√40 = 24.856, Mean ± SE: 88.68 ± 3.93 → Mean ± SD: 88.68 ± 24.86

**Somatostatin group, 1 years**

SD pretreatment eGFR = SE*√n = 4.23*√40 = 26.753, Mean ± SE: 77.86 ± 4.23 → Mean ± SD: 77.86 ± 26.753

**Control group, baseline**

SD pretreatment eGFR = SE*√n = 5.30*√39 = 18.360, Mean ± SE: 77.77 ± 5.30 → Mean ± SD: 77.77 ± 33.10

**Control group, 1 years**

SD posttreatment eGFR = SE*√n = 5.45*√39 = 34.035, Mean ± SE: 72.16 ± 5.45 → Mean ± SD: 72.16 ± 34.04

We assume the Correlation coefficient is same between 1 year and 3 years.

The Correlation coefficient at 3 years is calculated as followings.

Corr (EXP) = [SD_E(base)_^2^ + SD_E(final)_^2^ – SD_E(change)_^2^]/[(2*SD_E(base)_*SD_E(final)_] = (24.86^2^ + 29.47^2^ – 3.17^2^)/(2*24.86*29.47) = 1.007645939

Corr (CON) = [SD_E(base)_^2^ + SD_E(final)_^2^ – SD_E(change)_^2^]/[(2*SD_E(base)_*SD_E(final)_] = (18.36^2^ + 40.66^2^ – 4.13^2^)/(2*18.36*40.66) = 1.000612967

SD_E(change)_ = √[SD_E(base)_^2^+ SD_E(final)_^2^-(2*Corr*SD_E(base)_*SD_E(final)_)] = √(24.86^2^ + 26.75^2^ – 2*1.0*24.86*26.75) = 1.89

SD_C(change)_ = √[SD_C(base)_^2^+ SD_C(final)_^2^-(2*Corr*SD_C(base)_*SD_C(final)_)] = √(18.360^2^ + 22.551^2^ – 2*1.0*18.360*22.551) = 4.19

**Caroli (2013) 3 years**

**Somatostatin group, baseline**

SD pretreatment eGFR = SE*√n = 3.93*√40 = 24.856, Mean ± SE: 88.68 ± 3.93 → Mean ± SD: 88.68 ± 24.86

**Somatostatin group, 3 years**

SD posttreatment eGFR = SE*√n = 4.66*√40 = 29.472, Mean ± SE: 76.33 ± 4.66 → Mean ± SD: 76.33 ± 29.47

**Control group, baseline**

SD pretreatment eGFR = SE*√n = 5.30*√39 = 18.360, Mean ± SE: 77.77 ± 5.30 → Mean ± SD: 77.77 ± 18.36

**Control group, 3 years**

SD pretreatment eGFR = SE*√n = 6.51*√39 = 40.655, Mean ± SE: 64.64 ± 6.51 → Mean ± SD: 64.64 ± 40.66

**ΔeGFR (ml), Somatostatin group**

Median (IQR): -3.85 (-6.20～-1.92)

→　SD = IQR/1.35 = [-1.92-(-6.20)]/1.35 = 3.170, Mean ± SD: -3.85 ± 3.17

**ΔeGFR (ml) Control group**

Median (IQR): -4.95 (-7.49～-1.97)

→　SD = IQR/1.35 = [-1.92-(-7.49)]/1.35 = 4.126, Mean ± SD: -4.95 ± 4.13

Corr (EXP) = [SD_E(base)_^2^ + SD_E(final)_^2^ – SD_E(change)_^2^]/[(2*SD_E(base)_*SD_E(final)_] = (24.86^2^ + 29.47^2^ – 3.17^2^)/(2*24.86*29.47) = 1.007645939

Corr (CON) = [SD_E(base)_^2^ + SD_E(final)_^2^ – SD_E(change)_^2^]/[(2*SD_E(base)_*SD_E(final)_] = (18.36^2^ + 40.66^2^ – 4.13^2^)/(2*18.36*40.66) = 1.000612967

**Ruggenenti (2005)**

**Correlation coefficient**

Perico (2019)

Corr (EXP) = [SD_E(base)_^2^ + SD_E(final)_^2^ – SD_E(change)_^2^]/[(2*SD_E(base)_*SD_E(final)_] = (8.15^2^ + 6.07^2^ - 2.37^2^)/(2*8.15*6.07) = 0.986956873

Corr (CON) = [SD_C(base)_^2^ + SD_C(final)_^2^ – SD_C(change)_^2^]/[(2*SD_C(base)_*SD_C(final)_] = (11.70^2^ + 8.89^2^ - 2.81^2^)/(2*11.70*8.89) = 1.000

Caroli (2013)

Corr (EXP) = [SD_E(base)_^2^ + SD_E(final)_^2^ – SD_E(change)_^2^]/[(2*SD_E(base)_*SD_E(final)_] = (13.614^2^ + 16.143^2^ – 3.17^2^)/(2*13.614*16.143) = 0.901688935

Corr (CON) = [SD_E(base)_^2^ + SD_E(final)_^2^ – SD_E(change)_^2^]/[(2*SD_E(base)_*SD_E(final)_] = (18.360^2^ + 22.551^2^ – 4.13^2^)/(2*18.360*22.551) = 1.000612967

Hogan (2010)

Corr (EXP) = [SD_E(base)_^2^ + SD_E(final)_^2^ – SD_E(change)_^2^]/[(2*SD_E(base)_*SD_E(final)_] = (26.53^2^ + 25.66^2^ – 15.46^2^)/(2*26.53*25.66) = 0.825008248

Corr (CON) = [SD_E(base)_^2^ + SD_E(final)_^2^ – SD_E(change)_^2^]/[(2*SD_E(base)_*SD_E(final)_] = (28.08^2^ + 26.40^2^ – 13.21^2^)/(2*28.08*26.40) = 0.884204154

We assume the correlation coefficient for the study by Ruggenenti (2005) is the average correlation coefficient of above 3 studies.

Corr (EXP) = (0.986956873 + 0.901688935 + 0.825008248)/3 = 0.904551352

Corr (CON) = (1.000 + 1.000612967 + 0.884204154)/3 = 0.961605707

SD_E(change)_ = √[SD_E(base)_^2^+ SD_E(final)_^2^-(2*Corr*SD_E(base)_*SD_E(final)_)] = √(25.2^2^ + 23.6^2^ – 2*0.905*25.2*23.6) = 10.75

SD_C(change)_ = √[SD_C(base)_^2^+ SD_C(final)_^2^-(2*Corr*SD_C(base)_*SD_C(final)_)] = √(22.49^2^ + 25.7^2^ – 2*0.962*22.5*25.7) = 7.33
