# Supplementary material for: Somatostatin analog therapy effectiveness on the progression of polycystic kidney and liver disease: A systematic review and meta-analysis of randomized clinical trials
Source: PLoS One. 2021 Sep 24;16(9):e0257606. doi: 10.1371/journal.pone.0257606 (PMC8462725; doi:10.1371/journal.pone.0257606)
Supplement: S8 Table — (DOC) [file pone.0257606.s011.doc]

**(S8 Table) List of all adverse events**

|  | Hogan (2020) | Van Aerts (2019) | Perico (2019) ALADIN 2 | Meijer (2018) DIPAK 1 | Pisani (2016) | Caroli (2013) ALADIN | Hogan (2010) | Caroli (2010) | van Keimpema (2009) LOCKCYST | Ruggenenti (2005) |
| --- | --- | --- | --- | --- | --- | --- | --- | --- | --- | --- |
| Biliary commlications | | | | | | | | | | |
| Cholelithiasis |  | 1.1 / 0 |  | 0.7 / 0.0 | 7.1 / 0 | 5.0 / 0 | 3.6 / 0.0 |  |  | 8.3 / 0.0 |
| Cholecystitis |  |  |  |  | 7.1 / 0 | 5.0 / 0 |  |  |  |  |
| Gallbladder sludge |  |  |  |  |  |  | 3.6 / 0.0 |  |  |  |
| Alanine aminotransferase elevation |  |  |  |  |  |  |  |  |  | 16.7 / 8.3 |
| Hyperammonemia |  |  | 2.0 / 0.0 |  |  |  |  |  |  |  |
| Pancreatitis/Pnacreatic enzyme elevation |  |  | 2.0 / 2.0 | 0.7 / 0.0 |  |  |  |  |  |  |
| Gastrointestinal complications | | | | | | | | | | |
| abdominal pain, epigastric pain, Gastroenteritis | 3.4 / 0.0, 33.3 / 6.7 | 1.1 / 0 | 0.0 / 2.0 | 1.3 / 0.0 | 0 / 7.1 | 0.0 / 2.6 | 3.6 / 0.0 |  |  |  |
| Abdominal hernia/Umbilical hernia |  |  | 2.0 / 0.0 |  |  |  | 3.6 / 0.0 |  |  |  |
| Abdominal discomfort/Flatulence, abdominal cramping, gas |  |  |  | 97 / 20 |  |  | 50.0 / 21.4 |  | 59.3 / 0.0 |  |
| Conspitation |  |  |  |  |  |  |  |  | 3.7 / 0.0 |  |
| Abnormal feces (Diarrhea/Steatorrhea) | 51.5 / 53.3 |  |  | 91.0 / 6.6 |  |  | 64.3 / 28.6 |  | 70.3 / 11.1 | 25.0 / 0.0 |
| Pale stools |  |  |  |  |  |  |  |  | 40.7 / 0.0 |  |
| Biliary vomiting |  |  | 2.0 / 0.0 |  |  |  |  |  |  |  |
| Decreased appetite |  |  |  | 7.2 / 0.7 |  |  |  |  |  |  |
| Nausea | 27.3 / 20.0 |  |  | 29.0 / 4.6 |  |  |  |  |  |  |
| Ascites | 3.4 / 0.0 |  |  |  |  |  |  |  |  |  |
| Renal complications | | | | | | | | | | |
| Ureteral obstraction/Nephrolythiasis |  |  | 0.0 / 2.0 |  | 0 / 7.1 | 0.0 / 2.6 |  |  |  |  |
| Acute renal failure/Worsening renal disfunction |  |  | 3.9 / 4.1 |  |  | 0.0 / 2.6 |  |  |  |  |
| Hypertensive crisis |  |  |  |  | 0 / 7.1 | 0.0 / 2.6 |  |  |  |  |
| Cystocele |  |  | 2.0 / 0.0 |  |  |  |  |  |  |  |
| Genitourinary prolapse |  |  | 2.0 / 0.0 |  |  |  |  |  |  |  |
| Infections / fever | | | | | | | | | | |
| Hepatic cyst infection |  | 6.5 / 0 |  | 5.2 / 0 |  |  |  |  |  |  |
| Hepatic cyst hemorrage |  |  |  |  | 7.1 / 0 | 2.5 / 0.0 |  |  |  |  |
| Renal cyst infection |  | 2.2 / 2.4 | 2.0 / 6.1 | 2.0 / 2.0 |  |  |  |  |  |  |
| Urinary tract infection, pyelonephritis |  | 3.3 / 0 | 0.0 / 4.0 | 1.3 / 0.7 | 7.1 / 7.1 | 5.0 / 2.6 | 3.6 / 0.0 |  |  |  |
| Renal cyst hemorrage |  |  |  |  | 0 / 7.1 | 2.5 / 2.6 |  |  |  |  |
| Fever | 0.0 / 8.3 | 1.1 / 0 | 2.0 / 0.0 | 1.3 / 0.0 |  |  |  |  |  |  |
| Sepsis |  |  | 2.0 / 0.0 |  |  | 2.5 / 5.1 |  |  |  |  |
| Influenza-like illness |  |  |  | 20.0 / 30.0 |  |  |  |  |  |  |
| Varicella |  |  | 2.0 / 0.0 |  |  |  |  |  |  |  |
| HepatitisC |  |  |  |  |  | 0.0 / 2.6 |  |  |  |  |
| Rash |  |  |  |  |  |  |  |  | 7.4 / 0.0 |  |
| Nodules at injetion side |  |  |  | 5.9 / 0.0 |  |  |  |  | 48.1 / 0.0 |  |
| Injection side discomfort |  |  |  | 32.0 / 0.7 |  |  |  |  |  |  |
| Other complications | | | | | | | | | | |
| Hyperglycemia/Glycated hemoglobin increased | 78.8 / 26.7 |  |  | 5.2 / 0.7 |  |  | 10.0 / 2.0 |  |  | 0.0 / 8.3 |
| Diabetes | 59.4 / 6.7 |  |  |  |  |  |  |  |  |  |
|  |  |  |  |  |  |  |  |  |  |  |
| Fatigue | 39.4 / 20.0 |  |  | 42.0 / 21.0 |  |  |  |  |  |  |
| Anemia |  |  | 2.0 / 2.0 |  |  |  |  |  |  |  |
| Dizziness | 30.3 / 20.0 |  |  | 20.0 / 7.9 |  |  |  |  |  |  |
| Myocardial infarction |  |  | 0.0 / 2.0 |  |  |  |  |  |  |  |
| Chest pain |  |  |  | 7.8 / 1.3 |  |  |  |  |  |  |
| Pulmonary embolism |  |  | 2.0 / 0.0 |  |  |  |  |  |  |  |
| Arrhythmia/Bradycardia | 6.9 / 0.0, 30.3 / 6.7 |  |  | 15.0 / 5.9 |  |  | 3.6 / 0.0 |  |  |  |
|  |  |  |  |  |  |  |  |  |  |  |
| Headache | 15.2 / 20.0 |  |  |  |  |  |  |  |  |  |
| Intracranial aneurysm |  |  |  |  | 7.1 / 0 | 2.5 / 0.0 |  |  |  |  |
| Hair loss/Alopecia | 27.3 / 0 |  |  | 10.0 / 0.0 |  |  | 3.6 / 0.0 |  |  |  |
| Retinal detachment |  |  | 2.0 / 0.0 |  |  |  |  |  |  |  |
| Menometrorrhagia |  |  | 2.0 / 0.0 |  |  |  |  |  |  |  |
| Nasopharyngitis |  |  |  | 12.0 / 24.0 |  |  |  |  |  |  |
| Spinal colunm injury |  |  |  |  |  | 0.0 / 2.6 |  |  |  |  |
| Breast carcinoma |  |  |  |  |  |  |  |  | 0.0 / 3.7 |  |
| Neutropenia | 0.0 / 8.3 |  |  |  |  |  |  |  |  |  |
| Unspecified | 0.0 / 8.3 |  |  |  |  |  |  |  |  |  |

Black: serious adverse events, Red: mild adverse events
