# Supplementary material for: Somatostatin analog therapy effectiveness on the progression of polycystic kidney and liver disease: A systematic review and meta-analysis of randomized clinical trials
Source: PLoS One. 2021 Sep 24;16(9):e0257606. doi: 10.1371/journal.pone.0257606 (PMC8462725; doi:10.1371/journal.pone.0257606)
Supplement: S9 Table — (DOCX) [file pone.0257606.s012.docx]

**(S9 Table) Loss to Follow Up**

| **Study** | **n/N** | **Percentage of loss of follow up** |
| --- | --- | --- |
| Ruggenenti 2005 | 0/24 | 0% |
| Van Keimpema 2009 | 4/54 | 7.4% |
| Caroli 2010 | 0/24 | 0% |
| Hogan 2010 | 0/42 | 0% |
| Caroli 2013 | 9/79 | 11.4% |
| Pisani 2016 | 8/35 | 22.9% |
| Meijer 2018 | 44/305 | 14.4% |
| Perico 2019 | 30/100 | 30.0% |
| Van Aerts 2019 | 18/175 | 10.3% |
| Hogan 2020 | 7/48 | 14.6% |
