# Supplementary material for: Somatostatin analog therapy effectiveness on the progression of polycystic kidney and liver disease: A systematic review and meta-analysis of randomized clinical trials
Source: PLoS One. 2021 Sep 24;16(9):e0257606. doi: 10.1371/journal.pone.0257606 (PMC8462725; doi:10.1371/journal.pone.0257606)
Supplement: S10 Table — (DOCX) [file pone.0257606.s013.docx]

**(S10 Table) Hard outcome**

| **Author** | **Year** | **ESRD** | **Death** |
| --- | --- | --- | --- |
| Ruggenenti, P. | 2005 | 0 | 0 |
| van Keimpema, L. | 2009 | Unknown | 0 |
| Caroli, A. | 2010 | Unknown | 0 |
| Hogan, M. C. | 2010 | 0 | 0 |
| Caroli, A. | 2013 | Unknown | 0 |
| Pisani, A. | 2016 | Unknown | 0 |
| Meijer, | 2018 | Unknown | 1 |
| Perico | 2019 | 11 | 0 |
| Van Aerts | 2019 | Unknown | 1 |
| Hogan, M.C. | 2020 | 0 | 0 |
