# Supplementary material for: Somatostatin analog therapy effectiveness on the progression of polycystic kidney and liver disease: A systematic review and meta-analysis of randomized clinical trials
Source: PLoS One. 2021 Sep 24;16(9):e0257606. doi: 10.1371/journal.pone.0257606 (PMC8462725; doi:10.1371/journal.pone.0257606)
Supplement: Attachment — Submitted filename: Review Suwabe et al. PLOS one 22052021.docx [file pone.0257606.s014.docx]

In this well written systematic review and meta-analysis, Suwabe et al. summarizes the most important RCT’s that investigate the effectiveness of somatostatin analogues to slow progression of PKD and PLD. However, I think some of the information can be written or presented more clearly for the reader. Furthermore I miss some novelty of this review. Therefore I have a few suggestions for improvement.

**Major comments**

- The description on how the selection for the RCTs have been conducted is very clear. However, I miss in the methods what the authors exactly want to investigate and compare between the different trials in their meta-analysis. How do they define effectiveness? What are the most important outcomes? That only becomes clear in the result section. I think it would be more clear for the reader when the authors add more about this in their method section.
- I miss some speculation and discussion about some of the results in de discussion section. This research group is very specialized in PLD and somatostatin analogues and I am very curious about their thought about some of the results. This manuscript would be more novel when the authors add more speculation in their discussion section.

**Minor comments**

*Abstract*

- The authors mention in the result section first TKV and then TLV numbers. At last eGFR is mentioned. I would first describe TLV, then TKV and subsequently eGFR data. This is a more logical order because ofcourse TKV and eGFR are related.
- In the conclusion I miss a few words about ‘suggestions for the future’

*Introduction*

- The authors mention a good point about results with absolute volumes versus percentage change. This is very important and very often wrongly interpreted by some authors.

*Methods*

- The authors mention that they included RCTs that compared a somatostatin analogue versus placebo, although many of the included studies did not include a placebo group. Please change this wording.
- As mentioned in the major comments, I miss the description of which outcomes the authors want to investigate in their meta-analysis.

*Results*

- I understand that he authors want to say something about endpoints and somatostatin analogues. However, the data mentioned under ‘Hard outcomes’ is in my opinion not relevant. I think it would be better not mention this data here, but describe this in the discussion section for example. Also the authors should add some wording about what they think the reason is that there are no hard outcomes.
- Adverse events are presented as ‘change’; but change from what? Before and after starting somatostatin analogues? Or is this just a comparison between patients using somatostatin analogues and not using somatostatin analogues? In that case, change is in my opinion not the correct way to describe this data. Please only use percentage vs. percentage between two groups.
- The authors describe that they have performed several sensitivity analysis; do they mean that they only excluded studies with a cross-over design? First of all, isn’t that just one sensitivity analysis instead of several? Furthermore for me it is not quite clear why the authors perform this analysis; I think some additional wording should be added to explain this.

*Discussion*

- This is very minor, but I noticed two small typo’s:
  Sentence 227 ‘ We consider that **it** this should be’
  Sentence 250 ‘for patients whose age (<45) **that** those whose age’
- In sentence 253 the authors mention that young female patients seemed to have the most substantial effect of somatostatin analogues in a polled analysis. I miss here the explanation what the reason for this is. I am sure the authors are aware of de decrease in liver growth in women after menopause.
- Also very minor: Sentence 255 the authors ‘Rene *et al*’ are mentioned; this is his first name, his last name is ‘van Aerts’.
- In sentence 259 and on, the authors mention that they could not elucidate which subgroups could get the highest benefit from this therapy. Can the authors maybe speculate about this? Maybe young women are the ones who have the most benefit in term of absolute growth in liver volume? And maybe the fact that they do not need a liver transplant? The letter is also nowhere mentioned in this manuscript, although an important point.
- About adverse events of the somatostatin analogues, what do the authors think is the reason that they are better tolerated in PKD and PLD than in acromegaly and neuroendocrine tumors?
- In sentence 275 tolvaptan is suddenly mentioned. I ofcourse understand why tolvaptan is metioned, but it looks that it is mentioned here ‘out of the blue’. I think the authors should first begin that there is another therapy available, what it is, and then start to compare tolvaptan with somatostatin analogues. I think also important is maybe to compare adverse events om somatostatin analogues with tolvaptan. In my clinical experience somatostatin analogues are better tolerated than tolvaptan (but I believe existing data about this does not represent my experience).

*Conclusion*

- Here it is mentioned that somatostatin analogues slow progression of PKD and PLD; I think it would be better to mention here that there is only volume reduction…..

*Tables*

Table 1

- The title of the 5^th^ column is ‘Somatostatin’ the 6^th^ ‘Control’. Please adjust this to for example ‘Somatostatin therapy’ and ‘Control group’, to make this more clear.
- The 9^th^ column also mentiones ‘Control’ but there are no data for this column. Please delete this column.

Table 2

- There are no numbers for Post-treatment mentioned for ‘DIPAK 1’ and ‘van Aerts’. Is that data not available or is this by accident not filled out? Please clarify in the table when it is not available with for example putting *NA* in those fields.

Table 3

- As mentioned before, I would advise to only mention the percentages for each group instead of making a variable that represents change (which is in my opinion not suitable here).

*Figures*

Figure 2

- Also mention in the figures somatostatin therapy instead of only somatostatin to make it clear that you are talking about intervention versus control group.

Figure 3

- For the results of ‘van Aerts’ the forest plot shows a large green square and no 95% CI. This seems a bit odd. Is this an error in the figure?
